# Taking a closer look at matrix vesicle biogenesis

**DOI:** 10.1093/jbmr/zjaf076

**Published:** 2025-05-31

**Authors:** Charlotte Clews, Scott Dillon, Fabio Nudelman, Colin Farquharson, Louise A Stephen

**Affiliations:** Functional Genetics Division, The Roslin Institute and Royal (Dick) School of Veterinary Studies, University of Edinburgh, Midlothian EH25 9RG, United Kingdom; Yusuf Hamied Department of Chemistry, University of Cambridge, Cambridge CB2 1EW, United Kingdom; School of Chemistry, University of Edinburgh, Edinburgh EH9 3FJ, United Kingdom; Functional Genetics Division, The Roslin Institute and Royal (Dick) School of Veterinary Studies, University of Edinburgh, Midlothian EH25 9RG, United Kingdom; Functional Genetics Division, The Roslin Institute and Royal (Dick) School of Veterinary Studies, University of Edinburgh, Midlothian EH25 9RG, United Kingdom

**Keywords:** matrix vesicles, extracellular vesicles, exosomes, mineralization, calcification, osteoblast, chondrocyte, microscopy

## Abstract

Matrix vesicles (MVs) are crucial components in the development of a healthy mineralized skeleton. They are also the key elements leading to the pathological calcification of vasculature; however, we know surprisingly little about them. First characterized over 55 yr ago, MVs are small membrane bound extracellular vesicles (EVs) that provide an environment for the concentration of calcium and phosphate. This makes MVs the first step in producing calcium phosphate, leading to the nucleation of HA mineral within the extracellular matrix, the key process in biomineralization. In this review, we discuss the literature on MV biogenesis and explore their components in the context of EVs and exosomes. We consider MVs in light of the Minimal Information for Studies of Extracellular Vesicles (MISEV2023). In doing so, we identify striking parallels in the biogenesis, contents, and roles of MVs and exosomes, opening opportunities for new avenues of research and understanding. We also explore the debate around whether MVs really contain HA, and propose emerging technologies, particularly in the field of imaging, to improve our understanding of MVs and galvanize research in the area over the coming years. By taking a closer look at MV biogenesis, we will be able to make use of the emerging technologies around EVs more widely, with the aim of fully understanding these vital vesicles and harnessing their potential therapeutic benefits.

## Matrix vesicles: what are they and what do they do?

Matrix vesicles (MVs) are small, membrane bound extracellular vesicles (EVs) that concentrate minerals including calcium and inorganic phosphate (Pi), potentially transporting calcium phosphate to the collagen matrix. This allows for the nucleation of crystalline hydroxyapatite (HA), the mineral component of bone, within the extracellular matrix (ECM). This protein-mineral composite is stiff and tough, material properties that provide bone with flexibility, rigidity, and resistance to fracture.^1^^,^^2^ Recent studies into mineral formation and its incorporation into the ECM postulate more complex roles for MVs within the bone microenvironment.^3^^,^^4^ Meanwhile, our understanding of the biochemistry and physical chemistry that occurs within MVs to concentrate and control the generation of amorphous phases is incomplete.^3^ Nearly 60 yr after they were first identified by Bonucci and Anderson, the process of MV biogenesis and subsequent release from the osteoblast and chondrocyte is unclear and the topic of intense debate.^1^^,^^5^

**Figure 1:**
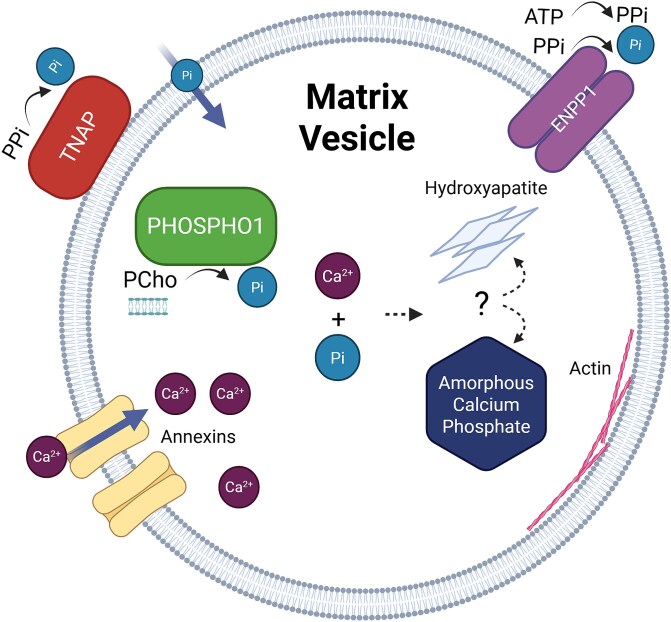
Schematic showing the current understanding of specific mineralization components of matrix vesicles (MV). Key proteins are orphan phosphatase 1 (PHOSPHO1), TNAP and ectonucleotide pyrophosphatase/phosphodiesterase 1 (ENPP1). Calcium is acquired via the actions of members from the annexin A family, where it combines with Pi in the MV lumen. The nature of the calcium-phosphate complex formed within the MV lumen is currently unknown but may be either amorphous or crystalline. Actin and actin related proteins are attached to the inner MV membrane possibly contributing to their motility in the matrix. Made with BioRender.

Matrix vesicles are released from the plasma membrane of osteoblasts and chondrocytes and contain a variety of molecules essential for bone mineralization. In particular, phospholipids, calcium channels, and annexins are crucial to their function, as are essential phosphatases such as tissue nonspecific alkaline phosphatase (TNAP) encoded by the *ALPL* gene, and orphan phosphatase 1 (PHOSPHO1).^6^^,^^7^ TNAP and PHOSPHO1 have nonredundant roles in the liberation of Pi from their respective substrates. In doing so, TNAP reduces the concentration of its substrate pyrophosphate (PPi), a potent inhibitor of mineralization produced from ATP by ectonucleotide pyrophosphatase/phosphodiesterase 1 (ENPP1).^8^^,^^9^ PHOSPHO1 is thought to act via hydrolysis of phosphocholine (PCho) and phosphoethanolamine derived from the vesicular membrane (summarized^7^). This synergistic balance maintains a Pi/PPi ratio permissive for normal bone mineralization, depicted in Figure 1.^6^ TNAP is an ectoenzyme bound to the surface of MVs through its GPI anchor, whereas PHOSPHO1 resides in the lumen of MVs.^10^ Deficiency of either phosphatase individually results in hypomineralized bone, whereas the absence of both phosphatases results in embryonic death and the complete absence of skeletal mineralization.^11^ The mechanism by which TNAP-generated Pi is transported from the extravesicular space to the MV lumen is unclear, but mice deficient in both PHOSPHO1 and the type III Na/Pi co-transporter (PiT-1/SLC20A1/Glvr1) have a more hypomineralized skeleton than that noted in animals lacking PHOSPHO1 alone, implicating the transporter in intravesicular Pi trafficking.^12^ Current hypotheses therefore postulate that MVs serve to generate the Pi required for bone mineralization in situ within the ECM through the synergistic action of TNAP and PHOSPHO1.

In contrast to the source of Pi in MV-mediated mineralization, the source of calcium is extensively debated in the field. In 1990, acidic phospholipid-dependent Ca^2+^-binding proteins (APD-CaBP), a group of annexin-related proteins, were reported to be responsible for the influx of calcium ions into the MV lumen, and further work has confirmed the presence and functional relevance of the Annexin A subfamily in MV-mediated mineralization.^13–15^ Nuclear magnetic resonance (NMR) spectroscopy has highlighted a potential role in the sequestering of calcium ions within MVs, for poly(ADP-ribose), a negatively charged polymer that complexes calcium ions into amorphous granules. Poly(ADP-ribose) has a defined binding site in collagen I fibrils and is abundant in mineralizing tissue.^16–18^ More recently, several groups have posited mitochondria as the hypothetical source of calcium.^19^^,^^20^

Once released into the extracellular space, MVs are anchored within the ECM and serve as nucleation sites for the growth and propagation of future mineral. However, the precise form of calcium phosphate that is present is unclear. Some studies show amorphous calcium phosphate (ACP) alone is present, while others suggest HA crystals may form within MVs.^21^^,^^22^ Due to the extremely high thermodynamic stability of HA, any accumulation of calcium and Pi is likely to induce crystal nucleation once a concentration barrier is reached. To maintain ACP as an amorphous phase, MVs would therefore need to control the physical chemistry of these ions through unknown mechanisms. One proposed mechanism is by complexing ions into metastable states through either chelation of calcium ions by charged molecules like poly-phosphorylated proteins, or poly(ADP-ribose), or by forming individual calcium phosphate complexes through interaction with components of the internal vesicle membrane, such as phosphoserine.^10^^,^^16^ Likewise, some sample processing protocols used to image MVs in which mineral HA has been identified have the potential to induce phase transitions due to dehydration. We must therefore consider the nature of ACP and HA in critiquing these studies and look toward methods less likely to induce ectopic mineralization to further understand the contents of MVs in vivo.

The mechanisms responsible for the anchoring of MVs to the bone matrix are unclear, but it is likely to involve roles for integrins, TNAP, and annexins.^12^^,^^23^^,^^24^ The MV membrane eventually breaks down, releasing its contents onto and within the collagen fibrils to mineralize the matrix.^25^^,^^26^ The mechanisms responsible for exposing ACP or preformed HA to the extracellular fluid, allowing for propagation of mineral along and within the collagen fibrils, are unclear. Perforation of the MV membrane by the mineral crystals may be involved, but a role for phospholipases and proteases in destabilization of the membrane has also been proposed.^24^^,^^27^^,^^28^ The process by which collagen fibril mineralization is regulated both temporally and spatially is also unclear, but may involve the deposition of ACP within the gap region of the collagen fibrils, where it transforms into HA crystals.^29^^,^^30^ This process is believed to be directed by highly acidic non-collagenous proteins.^31^^,^^32^

The origins of MVs and their biogenesis have not been extensively studied. Different groups have taken different approaches to their identification, focusing either on the cell and tissue of origin or on their contents. In this review, we propose that MVs cover all vesicles released by cells that contain calcium and phosphate and lead to the mineralization of ECM. We will discuss the biogenesis of such vesicles and will consider calcium-enriched intracellular vesicles (IVs) to be likely precursors to MVs. There are likely further outcomes for calcium-containing IVs, but at the time of writing, the literature does not lend itself to understanding what percentage of these IVs will become MVs. This review will describe our current knowledge on the formation of MVs in normal physiological conditions and specifically, we review the literature around their biogenesis, composition, and their role in skeletal development and biomineralization in the context of what is known about other EVs.

## EVs: a model for studying MVs

Extracellular vesicle is a catch-all term referring to an array of membrane-bound vesicles containing bioactive molecules destined for release by the cell, summarized in Table 1. EVs play vital roles in cell-to-cell communication, nucleic acid delivery, and various other functions, allowing signaling pathways to be enacted and the extracellular environment to be modulated.^37^^,^^38^

**Table 1:** Extracellular vesicles: biogenesis and function.

**Extracellular vesicle type**	**Size range (nm)**	**Biogenesis and function**
**Apoptotic bodies**	500-5000	Formed during apoptosis. Release cellular contents in a controlled manner, and act as signals for immune cells^33^
**Exopher**	1000-5000	Involved in the export of large cellular contents, including toxic proteins and whole organelles, during stress or damage^34^
**Ectosome**	100-1000	Budding vesicles released from the plasma membrane; involved in immune response, cell-cell communication, and cargo transport^35^
**Exosome**	30-150	Originate from multivesicular bodies (MVBs) as inter-luminal vesicles (ILVs) before being released into the ECM as exosomes. Involved in intercellular communication, waste disposal, and immune modulation^36^
**Matrix vesicle**	~50-300	Formed by cells in mineralizing tissue via a currently unknown mechanism. They concentrate minerals, including calcium and phosphate, for the bio-mineralization of ECM, critical for bone and cartilage formation^24^

The role of EVs in the mineralization of the ECM, particularly in bone and cartilage, is the prime focus of this review. While MVs have been studied for more than half a century, it is only in recent years that the EV field has come together to standardize the way we study EVs.^1^^,^^37^ The development of a standardized protocol for studying EVs makes this an opportune moment in time for reconsidering our understanding of MVs in bone. EVs come in a range of sizes, from small liposomes measuring around 20-30 nm, to apoptotic bodies exceeding 10 000 nm in diameter. They carry a diverse cargo, including proteins, nucleic acids, lipids, metabolites, and even organelles from the parent cell.^39^ Most EVs are smaller than 200 nm and have traditionally been classified into distinct subsets, for example, exosomes and ectosomes/microvesicles, based on size, cargo, function, or biogenesis.^40^ While for many years, MVs have been studied as a unique entity, there is now a growing body of evidence that advocates that these vesicles should be studied under the umbrella of EVs. In this section, we will discuss the roles and biogenesis of EVs, that they might provide greater insight into the actions and biogenesis of MVs.

First posited in 1946,^41^ EVs were initially studied in the late 1960s in human plasma, while ultracentrifugation and electron microscopy (EM) revealed membranous structures in the flagellated alga *Ochromonas danica.*^42^ Shortly thereafter, it was found that these vesicles could transfer nucleic acids, such as RNA, between cells. In 1981, the terms “exosome” and “microvesicles” were introduced to describe vesicles derived from the plasma membrane, often sharing specific membrane components with the parent cell.^43^ Two distinct populations of vesicles were identified by EM. The first has irregular shapes and diameters ranging from 500 to 1000 nm. Another characterized by a larger membrane-limited body containing smaller spherical vesicles around 40 nm was later recognized as the “multivesicular body” (MVB).^43^^,^^44^ Similar to MVBs, exophers are relatively large EVs known for encapsulating and degrading defective mitochondria to maintain optimal metabolic function.^45^ Additionally, some EVs can be produced via cytoskeletal extensions, such as filopodia-derived vesicles formed by Rac1 and I-BAR protein family-mediated scission, and ciliary ectosomes, which play roles in cell-cell communication and ECM remodeling.^46–48^

The nomenclature of EVs is an evolving story, and their current terminology has recently been summarized by Couch and colleagues.^49^ The work of the International Society for Extracellular Vesicles (ISEV) has been crucial in the 21st century for pushing for standardization of nomenclature and biological parameters of EVs.^37^ The authors recognize that, particularly in the field of MVs, where the particles of interest do not fit neatly into previously ascribed groups, the move toward standardization of experimentation will be vital in offering the framework required to fully understand MVs. Within this review, we have made every effort to follow the guidelines of MISEV2023, while appreciating that a lot of the research preceding this has taken place without the context of these guidelines. To describe the different routes of biogenesis and cargoes found in EVs, we will refer to commonly-used non-MISEV nomenclature, such as exosomes and ectosomes, while recognizing the need to move away from these terms as distinctions.^37^

### Biogenesis of EVs

Within the discussion of mineralization and MVs, two main pathways require attention: exosomal and ectosomal biogenesis. Exosomes are traditionally distinguished from ectosomes in that intracellularly, they are contained within an MVB, produced by an invagination of the plasma membrane.^50^ These MVBs contain intraluminal vesicles (ILVs), approximately 50-300 nm, which are so named as their formation includes budding inwardly into the MVB lumen and are the precursors to the eventual extracellular exosomes.^51^ To this day, the core physiological role of exosomes is still largely unclear; while it is believed that at least one function is to expel excess or unnecessary proteins and cell components, and maintain homeostasis, a more specific and targeted function for exosomes in cell-cell communication is emerging.^52^ Key cargoes and functions of exosomes are summarized in Table 2.

**Table 2:** Exosome cargoes and key functions.

**Cargo types**		**Function**
**Proteins**	Adhesion molecules, cytoskeleton, cytokines, ribosomal proteins, growth factors	Docking to the ECM, interior vesicle ultrastructure, regulation of the immune response, removing excess proteins, and cell-cell communication^52^
**Lipids**	Cholesterol, lipid rafts, and ceramides	Leftover molecules from exosome biogenesis, removal of excess membrane lipids^53^
**Nucleic acids**	DNA, mRNA, and miRNA	Promoting/limiting viral infection, cancer progression, cell neoplasia, regulation of recipient cell gene expression^52^^,^^54^

**Figure 2:**
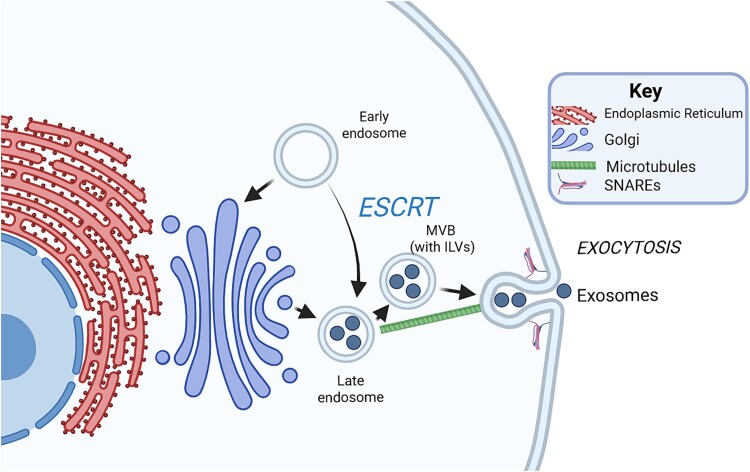
Relevant elements of the two main mechanisms for trafficking of multivesicular bodies originating from the trans-Golgi network, a key process in exosome biogenesis and release. ESCRT drives the formation of ILVs, and the selection and sorting of their cargo, giving rise to MVBs. These MVBs can be trafficked to the plasma membrane via the cytoskeleton, where they fuse with the help of docking proteins such as SNAREs, or fuse with lysosomes/autophagosomes to enable lytic degradation of their contents. Made with BioRender.

During the maturation of MVBs, various cellular components, including proteins, lipids, and nucleic acids, are selectively sorted into the ILVs. This sorting process, and the formation of ILVs from scission of the MVB membrane, is regulated by specific proteins such as endosomal sorting complexes required for transport machinery, sphingolipids, and accessory proteins (Figure 2^55–57^). Cargo molecules destined for secretion are included in this sorting, and the type of cargo determines the destination and fate of the vesicle populations; it is hypothesized that this sorting mechanism may be most reflective of the intracellular processing and packaging of mineralizing MV precursor vesicles.^53^

Once the cargo is sorted into ILVs within the MVBs, these MVBs can follow 2 main pathways. First, if the cargo is destined for destruction, these MVBs can fuse with lysosomes, leading to the degradation of their contents. The lysosomal degradation system of the cell degrades and disposes of long-lived proteins, aggregates, and damaged organelles, whereas the proteosome handles soluble, monomeric, likely misfolded proteins.^58–61^

Alternatively, for molecules that will be trafficked to extracellular sites, MVBs can fuse with the plasma membrane, releasing their ILVs into the extracellular space. Upon fusion with the plasma membrane, MVBs interact with various proteins, such as soluble N-ethylmaleimide–sensitive factor attachment protein receptor (SNARE) complex, which leads to the release of the ILVs contained within the MVBs into the extracellular space as exosomes.^62^ Briefly, some components of the motor assembly located in the vesicle membrane (v-SNAREs) intertwine with others in the target membrane (t-SNAREs), pulling the opposing membranes close together until they merge, releasing the cargo from the cell.^62^^,^^63^ This release can occur constitutively or in response to various stimuli or cellular signals. Once released, EVs can travel via Brownian motion (smallest EVs) or using matrix metalloproteinase mediated ECM degradation (larger EVs) to reach their destination.^64^ EVs are targeted to the cells within the ECM via surface proteins that bind to either ECM components, such as proteoglycans and glycoproteins, or plasma membrane proteins on the surface of recipient cell membranes.^65^ The EVs themselves, or their contents, can be taken up by these recipient cells through various mechanisms. In particular, SNAREs, Rab, and Sec1/Munc-18 related proteins have been associated with membrane fusion-based uptake; whereas phagocytosis, clathrin- and caveolin-mediated endocytosis, lipid rafts, and micropinocytosis are associated with the uptake of EVs by the recipient cell. The precise mechanisms associated with each cargo likely depend on the membrane composition of both EV and the target cell.^51^^,^^66^ In this way, EVs can influence various cellular processes such as gene expression, signaling pathways, and immune responses. This communication between cells mediated by exosomes is critical for physiological processes, including development, tissue homeostasis, and immune regulation, as well as pathological conditions, such as cancer, neurodegenerative diseases, and infectious diseases. In addition to these roles in cellular communication, many EVs are destined for roles in the ECM. In particular, we might consider the deposition of calcium phosphate and/or HA from MVs. Following release from the parent cell, these EVs are targeted not to cell membranes, but to the collagenous matrix, where they can modulate the structure and composition of the ECM (reviewed^67^).

The second type of biogenesis associated with mineralization of the ECM is produced by the outward budding of the plasma membrane; these vesicles are often referred to as ectosomes. Ectosomes are a heterogeneous vesicle type, carrying a range of cargoes, depending on the cell of origin. This includes apoptotic components as well as other bioactive proteins, lipids, and/or nucleic acids.^68^ The classic early MV observations from Anderson described budding of MVs from osteoblast plasma membrane protrusions, making this method of biogenesis particularly pertinent to the mineralization field.^69^ While subsequent studies have noted a potential exosomal, MVB pathway for MV biogenesis, it seems likely that at least some MVs are released by an ectosomal, budding mechanism.^69^^,^^70^

## EVs: key players in bone mineralization

In the previous sections, we have reviewed the various routes by which EVs are formed and how, upon delivery, their cargo mediates effective cell–cell communication and signaling within tissues. As a dynamic, responsive tissue, this is particularly true of bone. EVs released by osteoblasts, osteocytes, osteoclasts, and chondrocytes have been postulated to have many functions within the bone microenvironment, including ion exchange and ECM production to meet the specific demands of the body.^67^ Due to the variety of vesicles produced by cells, distinguishing those specifically involved in matrix mineralization from bone cell-derived EVs with other functions is challenging. However, without utilizing a method to detect ions or minerals within these vesicles or identify key MV proteins, they may be morphologically indistinguishable from other vesicle populations in the ECM. For example, some bone EVs have been shown to contain non-collagenous matrix proteins, such as bone sialoprotein, osteopontin, osteocalcin, and osteonectin, within the mineralizing bone environment.^71^ mRNAs mostly involved in bone-derived EV transcriptional regulation, such as BDP1, TAF7 L, and SOX11, and kinase activity (LPAR1 and ZEB2) are also found within these vesicles.^68^ MicroRNAs, such as miR-24, let-7, miR-143-3p, miR-10b-5p, miR-199b, miR-218, and miR-214-3p, have also been located within EVs formed from a variety of cells within bone and cartilage tissue. These microRNAs have important roles in the control of osteoblast and chondrocyte differentiation and therefore contribute to communication between bone cells.^72–74^ MVs isolated from the osteoblast cell line, MC3T3 E1 have also been shown to inhibit osteoclastogenesis via miR-125b, and this microRNA has been shown to protect against bone loss in murine disease models.^75^

Furthermore, matrix-bound nanovesicles (MBVs) may play a crucial role in the maintenance and mineralization of the ECM. These vesicles are anchored within the ECM and serve as vital biological signaling agents within this environment.^76^ Unlike exosomes or ectosomes/microvesicles, MBVs do not contain typical markers, such as CD63, CD9, CD81, or HSP70, suggesting a distinct biogenesis pathway.^77^ Moreover, MBVs exhibit tissue-specific characteristics, with unique lipid, RNA, and protein profiles possibly indicating novel and as yet unknown roles in ECM biosynthesis. They, however, do not share the same protein expression profiles as calcifying MVs.^78^^,^^79^

When discussing the investigation of calcifying MVs, it is crucial to consider the role of pH. Several studies have identified the importance of a narrow range of pH in allowing physiological mineralization of the ECM. Unsurprisingly, this is in keeping with the normal healthy pH of blood and interstitial fluid (pH 7.1-7.6).^80^ Acidosis resulting in changes as slight as a reduction to pH 6.9 is enough to alter osteoblast function via gene expression, as well as reducing mineralization of the ECM in ex vivo cell culture.^80^^,^^81^ This is likely due to reduced TNAP activity alongside an increase in Matrix Gla Protein, an inhibitor of mineralization, and a 2- to 4-fold increase in HA solubility in these conditions.^80^ Due to the range of physiological changes associated with changes in pH, ISEV recommends referring to pH when reporting on EVs.^37^ Throughout this review, where possible, pH is reported if in excess of normal physiological conditions.

### Biogenesis of MVs

Matrix vesicles studies have until now focused heavily on their contents rather than the route underlying their biogenesis. As discussed in the MISEV2023 guidelines, understanding vesicles based solely on their contents results in a loss of information on the potential roles of these vesicles.^37^ In understanding the biogenesis of MVs, we will better understand their biological makeup, as well as the wider roles they may play in ECM modulation and bone development. Such knowledge may unlock therapeutic opportunities to promote the delivery of MVs and their cargo to the collagen fibrils within the ECM to correct pathologies of mineralization.

Several studies promote the model of an MV precursor, where mineral components from the cell’s donor organelles are transported to the cell membrane, packaged into an MV, and released by the osteoblast or chondrocyte via an MVB-mediated pathway in the manner of exosomes.^4^^,^^44^ Understanding these “precursors” is vital to understanding the biogenesis of MVs. However, in accordance with MISEV2023 guidelines, we wish to avoid describing these particles based on a presumed extracellular role. For the purposes of this review, we are describing the biogenesis of calcium-enriched IVs. We cannot know, due to the constraints of current research, whether all calcium-enriched IVs are destined to be MVs. To understand the journey of these IVs, in-depth observation would be required, using additional markers of future MVs to visualize their outcomes. The following articles use either MVs isolated at a pH considered neutral (between 6.9 and 7.6) or visualized in vivo using cryo EM methods to maintain the integrity of the subcellular composition.

Mahamid et al. reported disordered mineral packets within IVs of bone lining cells within mouse calvaria and long bones studied using cryo EM.^19^ Proteomic analysis of MVs has identified a strong enrichment for proteins required for the packaging of cargo into ILVs in the exosome pathway.^4^^,^^82^ Similarly, Wuthier et al. have confirmed a phospholipid signature in MVs that resembles that of exosomes.^83^ Conversely, strong representation of filamentous actin and actin-related proteins identified inside the lumen of isolated MVs by Muhlrad et al. suggest an ectosomal release process, or may simply fulfill a requirement for the maintenance of a spherical form.^15^^,^^84–86^ Skelton et al. have used EV-specific dyes to identify exosome-like characteristics in MVs, particularly the regulated accumulation of RNAs within MVs and the endocytosis of MVs by MG-63 osteoblastic-like cells.^3^^,^^87^ These observations challenge the idea that MVs are solely a site for calcium phosphate accumulation and, rather intriguingly, suggest that they may have additional roles to play in the regulation of bone and cartilage physiology. While this is in contrast to the classic MV studies of Anderson and Bonnuci, depicting “blebbing” ectosomal EVs,^1^ an argument for both of these pathways to work in parallel is presented by Boonrungsiman et al.^1^^,^^20^ Using an anhydrous freezing substitution EM protocol, they visualize calcium-enriched IVs in murine growth plate samples, undergoing both intracellular (exosomal) and extracellular (ectosomal) biogenesis pathways.^20^ It is important to note that even those studies supporting the MV exosome-biogenesis model recognize that MVs are at the very least a highly specialized exosome, with specific RNA profiles and membrane compositions.^4^^,^^87^

### Mineral enrichment of MVs

The key role for MVs is to concentrate calcium and Pi, thus providing a mechanism for the delivery of ACP or HA to the ECM to mineralize cartilage and bone. While the phosphatases TNAP and PHOSPHO1 have been extensively studied in their roles to concentrate Pi, the mechanisms by which calcium is provided to the MV are as yet unclear.^88^

#### Endoplasmic reticulum as a source of mineral in MVs

**Figure 3:**
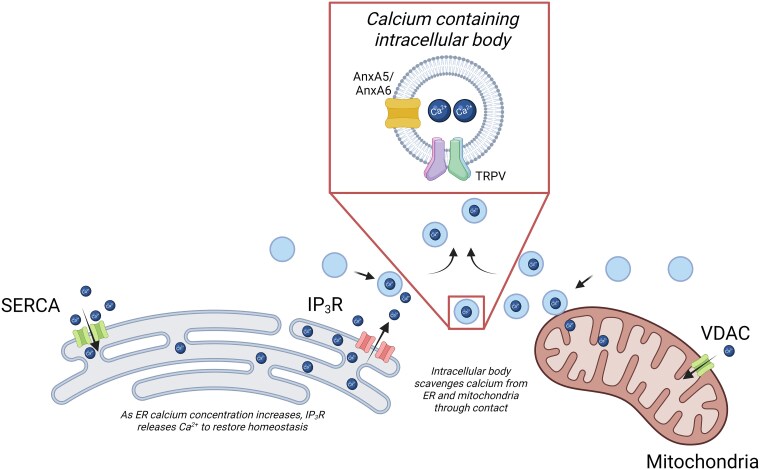
A summary of key players in endoplasmic reticulum (ER) calcium homeostasis and key proteins involved in the influx and efflux to other subcellular compartments. Calcium ions are moved into the ER via Sarco-/endoplasmic reticulum Ca-ATPase (SERCA) pump through ATP hydrolysis into ADP and Pi. This increases the ER calcium concentration. To transport calcium from the ER lumen, inositol 1,4,5-trisphosphate receptors (IP_3_Rs) are activated (via calcium) and cause release into mitochondria or lysosomes as required, via a voltage-dependent anion channel. Vesicles can also be receivers of this calcium, a process mediated by transient receptor potential cation channels and annexins. AnxA5 and AnxA6 are annexins thought to be present in the membrane of matrix vesicles. Made with BioRender.

The ER is well recognized as a key organelle in protein production and is highly developed in mineralizing cells, due to their high protein production capacity. It is also crucially a key store of most, if not all, intracellular calcium, maintaining ionic balance within the cytoplasm, and transporting calcium to mitochondria, lysosomes, endosomes, and other secretory vesicles.^89^^,^^90^ This process is summarized in Figure 3.

The ER also performs a crucial role in the formation of endosomes, lysosomes, ILVs within the wider MVB, and other vesicular cellular components, providing a logical origin for not only calcium ions but also for calcium-enriched IVs.^91^ The ER’s network of tubules and saccules maintains high calcium ion concentrations (up to millimolar levels when filled) through calcium ion influx from the systemic circulation.^92^ Key players in ER-mediated control of calcium ions are the sarco-/endoplasmic reticulum Ca-ATPase (SERCA) pump, which facilitates calcium entry to the ER, and inositol 1,4,5-trisphosphate receptors (IP_3_Rs), which mediate release of calcium from the ER and Golgi, allowing its redistribution to other organelles throughout the cell.^93–97^ When the ER’s calcium ion levels drop, the lumen is refilled via the actions of various calcium channels, including store-operated calcium entry channels and SERCA, among others.^97^^,^^98^ Likewise, when levels are too high, the ER then releases calcium ions, which are then absorbed by endosomes, lysosomes, or possibly MVs.

For all three IP_3_R subtypes, the binding of IP_3_ enables the channel to open, a process facilitated by calcium. This receptor has been widely studied in skeletal muscle and is required for the early development and function of neuromuscular junctions. However, at the time of writing, the mechanisms of calcium release from the ER and particularly its links to IV trafficking of calcium has yet to be studied in bone.^99–101^ Intracellular calcium dysregulation can induce ER stress, a feature that has been observed in some skeletal disorders, such as osteogenesis imperfecta, which is characterized by mineralization defects.^102–104^

Calbindins buffer cytosolic calcium ions and are critical for calcium transport and function throughout the cell.^105^^,^^106^ However, the entry of cytosolic calcium ions into ER vesicles is primarily driven by calcium-selective transient receptor potential cation channels and annexin channel proteins present in phospholipid membranes. The latter include AnxA5 and AnxA6, which have been found in MVs.^107^^,^^108^

#### Mitochondria as a source of mineral in MVs

Mitochondria, often referred to as “the powerhouse of the cell,” are in fact machines of phosphorylation, and highly implicated in the storage and trafficking of calcium and Pi. Whether mitochondria actively store calcium ions under normal physiological conditions is uncertain, but there is evidence for the sequestering of calcium phosphate granules by mitochondria both in vivo and in vitro*.*^20^^,^^109^^,^^110^ While Csordás et al. assert that mitochondria do not store calcium ions under physiological conditions, it does seem clear that they are at least capable of facilitating the uptake of cations, such as calcium, while also containing proteins responsive to changes in calcium levels in the matrix.^111^ Moreover, Boonrungsiman et al. have shown strong evidence for the presence of calcium phosphate granules within mitochondria of cultured osteoblasts, supporting multiple studies that have identified interactions between IVs and mitochondria.^70^ This accumulated knowledge lends itself to a mitochondrial-EV feedback loop whereby accumulation, storage, and export of calcium in osteoblasts are managed by mitochondrial interactions.^70^^,^^112^

#### Lysosomes as a source of mineral in MVs

Lysosomes are a key subcellular organelle with many roles in the maintenance of cell homeostasis, such as waste disposal and recycling, and the digestion of materials taken up from the ECM via endocytosis.^58^^,^^113^ Lysosomes are crucial in the act of exocytosis, due to their ability to reseal membranes, a process which is dependent on calcium ions and key lysosomal cysteine proteases cathepsin B and L.^114^ This means that any study extrapolating the colocalization of lysosomes with ECM components to explain intracellular processes must be considered in this context. While studies have suggested that lysosomes may act as intracellular MV precursors, there is perhaps more evidence for a role of lysosomes in calcium provision.^114^^,^^115^ Lysosomes have been shown to receive calcium ions from the ER via inositol receptor binding, and alongside acidocalcisomes, can serve as calcium shuttles in cells, providing an opportunity for calcium delivery to IVs.^114^ It is important to note that acidocalcisomes, small electron-dense membrane-bound acidic organelles, are rich not only in calcium ions but also polyphosphates, suggesting a calcium-phosphate transporter candidate for the intracellular movement of mineral precursors.^116^

## How we study MVs

There is still much to understand about MVs, their function, and how they are formed. As the fields of EVs and mineralization move forward, it is vital that we make the most of all the tools available to us. In the last few years, an explosion in new higher resolution imaging and isolation techniques will allow these studies to flourish, if used correctly. Furthermore, the unification of the EV field within ISEV and the associated guidelines offers an important framework in which to embed our studies.

### A closer look at imaging MVs

Studying EVs presents distinct challenges not typically encountered in traditional cell biology studies. This is primarily due to their small size and the diverse range of vesicle types produced by an equally diverse range of cells. Although EVs and many IVs perform specific and often vastly divergent functions, their morphology and size can be similar, making them difficult to differentiate from other vesicle populations. Despite these difficulties, specific vesicle dyes and key proteins known to associate with particular vesicle populations serve as reliable markers for identifying vesicles and, in some cases, tracking their movement from biogenesis to their functional destination.^117^^,^^118^

### EM and its contribution to imaging vesicles in bone

Measuring between 50 and 200 nm in diameter, MVs are typically too small for traditional fluorescence microscopy, making their imaging primarily reliant on EM. Initially identified by Bonnuci and Anderson using cutting-edge EM techniques, MVs have been studied in osteoblast mineralization and their role in vascular calcification through EM methods, such as transmission electron microscopy (TEM) and scanning electron microscopy (SEM).^1^^,^^5^^,^^119^ However, while room temperature EM techniques offer resolution of 0.1-1 nm, the process has some unavoidable issues, such as artifact introduction via fixation and dehydration, which are particularly problematic when attempting to observe delicate amorphous phases, which may crystallize depending on the method of preparation.^120^^,^^121^ Energy-dispersive X-ray spectroscopy or electron energy loss spectroscopy detectors are often added to the EM and are sufficient to establish the presence of calcium and Pi within MVs of mineralizing bone or cartilage.^122^^,^^123^ The main applications of EM in the study of MVs and bone formation are summarized in Table 3.

**Table 3:** Electron microscopy approaches appropriate to MV studies.

**Technique**	Method	Use in MV studies
**Transmission electron microscopy**	Transmits electrons through thin samples, provides high-resolution ultrastructure details of MVs, including their size, shape, and internal organization	Identification of IV subcellular localisation, particularly electron-dense calcium-enriched IVs. Identification of MVs at point of release
**Scanning electron microscopy**	Provides lower resolution, detailed images of surface topography	Identification of membrane-dependent methods of MV release, membranous extensions, already used to observe MVB objects^19^
**Cryo-focused ion beam-scanning electron microscopy (cryoFIB-SEM)**	Allows for 3D imaging of biological tissues like bone while preserving native tissue and intracellular structures	Analysis of IVs containing mineral precursors in bone-forming cells^124^

Alternative sample preparation techniques in EM aim to minimize artifacts. Cryo-EM, a specialized form of TEM, captures samples at cryogenic temperatures to preserve their structure, offering insights into MVs in their hydrated state.^19^^,^^125^ High-pressure freezing followed by freeze substitution has also been used to simultaneously preserve mineral and ECM within osteoblast cultures.^20^^,^^70^ These preparation techniques, while effective, require expensive equipment and expertise, so studies performing cryo-EM in native biological tissues to study the form and function of MVs have been limited. Atomic force microscopy, another high-resolution tool, provides nanoscale topographic images of MV surfaces, enabling studies of their mechanical properties and mineral progression.^120^

### Fluorescent microscopy to image MVs

Very few studies have utilized fluorescence microscopy to investigate MVs, due to the diffraction limit constraining the resolution of these techniques. However, the technology does have the potential to complement and build on the data obtained from existing ultrastructural studies.^126^ Considerable progression using this approach has opened up new, more robust opportunities for the imaging of small vesicles on the nanometer scale. The new generation of super-resolution microscopes offers the chance to study the intricacies of physical interactions between MVs and other cellular organelles and processes, both within the cell and the extracellular milieu. Their more intuitive user-interfaces make them a much more realistic option for researchers developing their imaging capabilities in this field.

Optical fluorescence microscopy encompasses a vast variety of current bioimaging, both in vitro and in vivo*,* using both living and fixed samples, and is essential to most areas of modern biological and biomedical research. Where EM can suffer from artifacts arising from sample preparation, fluorescence microscopy is limited by the numerical aperture of the objective and the diffraction limit of light: ~200 nm laterally and ~500 nm axially. Sample preparation and image processing can allow this diffraction limit to be bypassed, allowing exploration into yet undiscovered territory in terms of bioimaging.^127–129^

The main approaches available to achieve the required nanometer resolution to identify MVs and calcium-enriched IVs include stimulated emission depletion microscopy and single-molecule localization microscopy, as described in Table 4.^137^ Confocal imaging, enhanced by techniques like the Zeiss Airyscan, provides resolution down to ~120 nm, facilitating MV imaging with more widely available resources.^138^ Holotomography, a label-free imaging method, also enables 3D refractive index imaging of cells and tissues.^136^^,^^139^

**Table 4:** Fluorescent imaging approaches appropriate to MV studies.

**Technique**	Method	Resolution
**Stimulated emission depletion microscopy (STED)**	Super-resolution imaging based on two diffraction patterns, reducing surrounding noise^130^	~50 nm (~20 nm)
**MINISTED nanoscopy**	Precision of the STED technique is improved to Angstrom level by encircling the fluorophore with the low-intensity central region of the STED donut beam while constantly increasing the absolute power^131^	~1-2 nm
**MINimal photon FLUXes (MINFLUX)**	Combines the strengths of STED, PALM/STORM and other fluorescent “nanoscopies” to separate the fluorophores in the sample by activating and deactivating them individually per diffraction region, improving the resolution further^132^	~1-5 nm
**3D Structured illumination microscopy (SIM)**	Wide-field technique, using Moiré fringes and multiple images to identify the true fluorophore centre^133^	~300 nm
**Single molecule localization microscopy (SMLM)**	Wide-field illumination in combination with fluorophores that are stochastically switched between a long-lived off-state and a bright on-state^134^	20 nm
**Laser scanning confocal imaging with Airyscan processing (Zeiss)**	Traditional laser scanning confocal microscopes use a combination of pinhole and single-point detectors^135^	~120 nm
**Holotomography**	Holotomography (HT) is a quantitative, laser-based technique used to measure the three-dimensional refractive index (RI) tomogram of cells and tissues^136^	~1000 nm

In practice, due to limitations in antibody efficiency, understanding of MV constituents, and the non-specific nature of fluorescent dyes to all vesicle types, fluorescent imaging of MVs is a much greater task than it first seems. Calcium-based dyes, such as Calcein AM, and organelle markers, such as lysotracker, visualize intracellular movement of calcium within lysosomes of mineralizing cells and have been reported in MV studies,^3^^,^^70^ while vesicle membrane targets, such as phosphatidylserine and annexins, offer another route forward in visualizing MV biogenesis.^126^ Zebrafish transgenic reporter lines can also offer a fantastic opportunity to differentiate EVs of different cell origins.^140^

Recent techniques developed to increase the resolution of objects smaller than 100 nm include expansion microscopy (ExM). Structures within the cell can be accurately expanded using a hydrogel matrix. This is then expanded by a factor of 4-10 by hydration. However, due to the sample preparation protocol, which includes polymerization, digestion, and expansion, ExM is not live cell compatible and can distort the biological structure as a result of nonisotropic expansion.^141^ The suitability of ExM for use in bone and vesicles is yet to be fully explored, but in combination with the fluorescence techniques listed above, it offers the exciting potential to identify MV localization and distribution within the cell and in bone tissue.

### Correlative light and EM: the gold standard of MV imaging?

Correlative light and electron microscopy (CLEM) offers a perfect marriage between the high structural resolution of EM with the specificity of fluorescently tagged super resolution microscopy, whereby combining multiple imaging techniques, it is possible to obtain complementary information about the structure and composition of samples.^142^ Tissue samples are first processed for fluorescence light microscopy, and images of labeled proteins are obtained. The same sample is then processed and imaged via EM, allowing subcellular detail to be correlated with protein localisation.^143^ Several developments, including specific fluorescence/electron compatible markers for efficient correlation, better preservation of fluorescence, and integrated laser and electron microscope (iCLEM) technologies, have moved CLEM center-stage in the future of MV studies.^144^^,^^145^ This technique opens the possibility of identifying a fluorescent MV in a sea of other EVs, while also gaining nanometer-scale resolution of cellular ultrastructure and collagenous ECM.^126^^,^^146^

However, one of the major limitations of CLEM is the complex and challenging sample preparation process, to achieve an incredibly low throughput technique. To image the same samples for both light and electron microscopy, fixation, sectioning, and staining must be compatible with both techniques. This preparation, particularly using chemical fixation, can introduce artifacts that compromise the structural integrity of vesicles or cellular architecture, particularly small MVs. Switching to cryo-CLEM, where samples are high-pressure frozen and imaged under cryogenic conditions would eliminate this issue. The fast freezing ensures that cellular ultrastructure is preserved in a physiologically relevant matter,^147^ and the combination of fluorescence imaging and EM techniques such as cryo-focused ion beam scanning EM and cryo-transmission EM allows MVs to be studied in their biological context.^147^ However, this technique requires an extremely rapid transition between the light microscope, HPF, and EM, made easier by additional modules for such a function (such as the EMPACT2 and Rapid Transfer System developed by Leica Microsystems). Despite this, any manner of fixation sacrifices the ability to observe vesicle dynamics in real-time, thus limiting the study of vesicle populations during a live cellular process such as matrix mineralization.

Furthermore, the maximum resolution mismatch between Light and EM means that, while CLEM improves the final image resolution by combining these 2 modalities, the 2 techniques operate at vastly different scales, as discussed previously.^120^^,^^121^ This difference in resolution makes aligning images from both modalities difficult. Even slight misalignments between the datasets or a lack of sufficient data in the Z-plane can lead to incorrect interpretations of vesicle positions and interactions, undermining the accuracy of the findings. In addition to this, successfully integrating the data from both LM and EM can also be technically demanding. Aligning vesicle locations from fluorescence images with their high-resolution counterparts in electron micrographs requires either alternative visual aids (such as correlated brightfield images and stitched tile scans) and a good eye for pattern recognition, or specialized software tools for co-registration and image alignment, which can be time-consuming and computationally intensive. There are however many useful software tools with specific CLEM plugins which can streamline this process, such as the EC-CLEM plugin for image analysis software ICY.^148^ Despite these disadvantages, and if performed accurately on well prepared samples, CLEM has exciting potential to shed light on the biogenesis and constituents of specifically marked MVs in the contextual environment of a mineralizing ECM.

### Other technologies to help us understand MVs

As we interrogate the function and dynamics of MVs further, we must look to new technologies, not currently associated with the field. NMR, for example, uses the interaction of an external magnetic field with the nuclear spins of the sample to provide information about the local chemical environment of those nuclei. NMR has been extensively used to understand the detailed molecular structure of bone mineral.^149^ Work from several groups has revealed that bone mineral is comprised of thin (~1 to 2 nm) crystalline calcium phosphate platelets, surrounded by an amorphous hydrated Pi phase and strongly-bound water. These units are stacked, separated by symmetry-breaking metabolite ions, including citrate and lactate.^149–151^ Although NMR has yet to be applied directly in understanding MV biology, in solid-state experiments, samples can be interrogated in their native hydrated states, without the need for sample processing. This makes NMR an ideal approach for understanding the intricate biochemistry and physical chemistry occurring within MVs at atomic resolution, without disturbing delicate chemical equilibria. While NMR provides extremely high-resolution information, the length scales accessible prevent interrogation of long-range structure, and so offer a complementary technique in combination with the other technologies discussed in this review.

Isolation of MVs can be a vital tool in understanding their role in biomineralization using in vitro techniques.^152^ To isolate MVs, researchers have predominantly relied on ultracentrifugation to separate vesicles of the correct approximate size.^153^^,^^154^ A clear disadvantage of this technique is the inability to isolate different types of vesicles of a similar size; it is not clear what percentage of the isolates are true MVs. Utilizing many of the fluorescent markers discussed above, the EV field has embraced flow cytometry as a key technique allowing EVs to be selected based on their size and shape, as well as the markers expressed on their membranes.^155^ In the case of MVs, this could allow TNAP-positive MVs to be isolated more efficiently and with greater specificity from other EVs. In particular, the new generation of imaging flow cytometers allows for even greater precision and visualization of vesicles.^156^ The multiplexing afforded by flow cytometry could open a number of avenues for understanding the composition and biology of MVs. Furthermore, the recent development of the CAGS-Snorkel mouse model, by Monroe and colleagues, may allow for the isolation of tagged EVs from specific cell types in vivo to be used for a range of applications, including imaging, flow cytometry, co-culture, and omics.^157^ While discussing animal models available for the study of MV biogenesis, it would be remiss to omit zebrafish. Highly genetically amenable and with the benefit of a fully observable larval development, zebrafish bones have long been noted to undergo the same MV-driven mineralization process as mammalian bone.^140^^,^^158^^,^^159^ Furthermore, the zebrafish offers an external source of mineralizing tissue, the scales, and a recent slew of studies have shown the benefits of using zebrafish scales as a model for mammalian biomineralization.^159–161^ In combination with the imaging techniques we discuss above, these emerging mouse and fish models offer great opportunities to study MV biogenesis in cell culture, in vivo*,* and ex vivo*.*

## Concluding remarks

**Figure 4:**
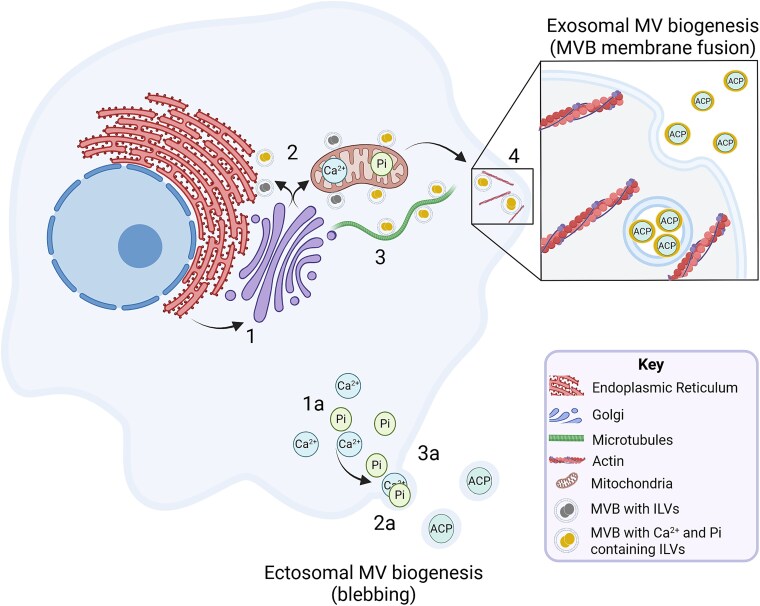
Matrix vesicle biogenesis. A schematic summary of the key steps in MV biogenesis as laid out in this review. There are two pathways implicated in biogenesis of MVs, exocytosis and ectosomal blebbing. Exocytosis follows these steps: (1) Production and packaging of MV components (phosphatases and other proteins) into vesicles by ER and Golgi, forming MVBs. (2) MVBs containing ILVs scavenge calcium and calcium/phosphate from the ER and mitochondria respectively. Ions are transported into the lumen of ILVs. (3) MVBs containing calcium/phosphate-enriched ILVs are trafficked along the microtubule cytoskeleton to the membrane periphery. (4) MVBs release calcium/phosphate enriched ILVs, now MVs, into the ECM via an exocytosis mechanism. The classic MV blebbing mechanism is proposed as follows: (1a) Calcium and phosphate within the cell is concentrated at the cell membrane. (2a) Cell membrane invaginations form microvesicles enriched with calcium and phosphate. (3a) Microvesicles are released into the ECM via an ectosomal mechanism. Made with BioRender.

Extracellular vesicles encompass a variety of membrane-bound compartments that deliver bioactive molecules and mineral ions from one cell to another, as well as to the ECM. MVs represent a specific subset of EVs that are critical for cartilage and bone mineralization. They provide a membrane-limited environment to concentrate calcium and Pi and deliver it in the form of ACP or HA crystals to the collagen fibrils of the ECM. However, the origins and key functions of MVs remain a topic for intense debate. Since the pioneering studies of Anderson and Bonucci in the late 1960’s it has been accepted that MVs are formed from blebbing of the osteoblast and chondrocyte membrane. Through the actions of phosphatases and annexins, these MVs accumulate Pi and calcium, the basis for HA formation within the collagen-rich ECM. While mineral has been reported within the MV, the question of how these mature crystals might lead to the propagation of crystals within the ECM remains unanswered. Modern approaches to studying MVs and the identification of mineralized granules within IVs and mitochondria suggest a likely contribution of exosomal biogenesis to MV development. Furthermore, optimum sample preparation offering improvements in preservation of cell structure and mineral composition and crystallinity has allowed us to investigate the form of the mineral within these intra- and extracellular MVs, suggesting the classic hypothesis of HA within the MV may not be as certain as previously believed.^20^^,^^124^ We summarize the findings of this review in Figure 4.

This debate tends to focus on whether MVs are a distinct subset of EVs or part of a broader population of exosomes due to their similar size, shape, and suspected methods of biogenesis and ECM release.^4^ This ambiguity, coupled with varying vesicle isolation methods, can result in isolated “MV” populations being contaminated with other similar vesicle types, leading to less conclusive experimental results. Additionally, few studies have examined MVs in the context of the guidelines provided by ISEV. This is partly due to the relatively recent expansion of the vesicle field, which has necessitated more rigorous experimental standards to which older MV work has not been subjected.^37^ Given the numerous potential applications of EVs in research and therapeutics, ensuring sufficient quality control in isolation and characterization is crucial for future MV studies. With the explosion of new techniques to aid in the study of EVs and IVs, now is the perfect time for the bone and mineralization community to join with the EV community, akin to the historical MV research that has benefited from an amalgamation of advances in the fields of biology, chemistry, and physics.

Understanding MVs continues to be vital in progressing our understanding of biomineralization and in the treatment of both hypo- and hyper-mineralized pathologies. To unpick the biogenesis and functions of these nanometer-sized vesicles, the field must embrace developing technologies in imaging while understanding MVs within the wider EV environment. As interest in cell-derived vesicles grows, we can expect to see answers to key questions about MV biogenesis and their mechanisms of mineralization. However, caution should be taken when choosing the most appropriate methods to image and define these vesicles, and the subsequent analysis to be performed within an EV population. With this in mind, combined with increased diligence in vesicle isolation and analysis methods, researchers may finally be able to tie together decades of bone research to answer the longstanding questions about MVs: where do they come from, and where do they go?

## Data Availability

No new data were generated or analyzed in support of this research.
